# Polypharmacy in a Patient With Intellectual and Developmental Disabilities

**DOI:** 10.7759/cureus.22019

**Published:** 2022-02-08

**Authors:** Rishi Raj, Douglas Owen, Lakshmi Kannan, Ummerubab Syeda

**Affiliations:** 1 Endocrinology, Diabetes and Metabolism, Pikeville Medical Center, Pikeville, USA; 2 Internal Medicine, University of Pikeville-Kentucky College of Osteopathic Medicine, Pikeville, USA; 3 Nephrology, Pikeville Medical Center, Pikeville, USA

**Keywords:** prader-willi syndrome, drugs, intellectulaly and developmental disabilities, acute kidney injury, polypharmacy

## Abstract

Prader-Willi syndrome (PWS) is an uncommon condition and its clinical manifestation in adulthood includes central obesity, hypogonadism, osteoporosis, cardiovascular disease, diabetes mellitus, and sleep apnea. These patients often have mild to moderate intellectual disability and are dependent upon their caregiver for healthcare needs. Hence, they may be at increased risk of polypharmacy-related complications, if there is poor communication between healthcare providers and caregivers. We present a case of a 26-year-old adult with PWS and mild to moderate intellectual disability, who was found to have acute kidney injury resulting from drug interaction between multiple nephrotoxic medications. Our case report highlights the importance of continuity of care with primary care providers, especially in patients with intellectual and developmental disabilities (IDD).

## Introduction

Adults with intellectual and developmental disabilities (IDD) constitute about 2%-3% of the population in the United States of America [1,2]. These patients often have multiple associated comorbidities and hence are on multiple prescription medications, resulting in polypharmacy-related adverse events [3]. Additionally, the prevalence and severity of polypharmacy-related adverse events are greater among patients with IDD compared to those without IDD [2]. With rising rates of polypharmacy among young adults, there is a significant chance of more adults with IDD having polypharmacy-related adverse events. With our case report, we want to focus on these potentially preventable adverse events among adults with IDD.

## Case presentation

A 26-year-old White woman with a medical history of PWS with mild to moderate intellectual disability, type 2 diabetes, congestive heart failure, obstructive sleep apnea, and pulmonary hypertension presented to the emergency room with watery diarrhea, generalized weakness, and bilateral lower extremity swelling for two days. Before the presentation, she was evaluated at the primary care physician's office for the above symptoms and had abnormal kidney function tests. She also had a recent history of acute otitis media with effusion, treated with Cefdinir for five days. There was no history of dysuria, cough, fever, or chills. The patient's former primary care doctor retired a few years back, and multiple locum tenens physicians provided medical care to the patient. Her home medications included: citalopram, levocetirizine, torsemide, potassium chloride, insulin glargine, sitagliptin, rosuvastatin, isosorbide mononitrate, chlorthalidone, enalapril, and valsartan. Additionally, her medication regimen had been changed significantly in the past six months to one year. On presentation to the emergency room, she was tachycardic (110 beats per minute), normotensive (111/80 mmHg), and was saturating 100 % on 3L nasal cannula. The rest of her physical examination was significant for increased body habitus, bilateral pitting edema, and short stature. Laboratory workup revealed elevated blood urea nitrogen (BUN), creatinine, and reduced estimated glomerular filtration rate (eGFR) along with hyperkalemia. The rest of the laboratory workup is summarized in Table 1. Urinalysis was negative for protein or RBCs. The renal ultrasound was normal. The patient was diagnosed with acute kidney injury (AKI) with hyperkalemia secondary to polypharmacy. Potentially nephrotoxic medications (e.g. enalapril, chlorthalidone, valsartan, metformin, and sitagliptin) were discontinued, and others were held based on her renal function. Hyperkalemia was acutely treated with insulin and dextrose, followed by a potassium binder. The patient also underwent gentle hydration and one dose of intravenous furosemide with significant improvement in renal function tests. Two days following admission, her renal function improved to BUN 71 mg/dL, creatinine 1.2 mg/dL, potassium 3.8 mmol/L, and eGFR 54.3 ml/min/1.73m^2^. 

**Table 1:** Laboratory workup on admission

Laboratory test	Results	Reference range
White blood cells (WBCs)	9.9	3.0-11.0 K/mm^3^
Hemoglobin (Hb)	9.2	13.0-18.0 g/dL
Hematocrit (HCT)	29.1	39.0-52.0%
Platelets	307	120-450 K/mm^3^
Blood urea nitrogen (BUN)	141	6-24 mg/dL
Creatinine	3.4	0.6-1.2 mg/dL
Estimated glomerular filtration rate (EGFR)	16.3	>59 mL/min
Sodium	134	133-144 mmol/L
Potassium	6.3	3.6-5.2 mmol/L
Chloride	105	96-106 mmol/L
Calcium	9.8	8.6-10.3 mg/dL
Bicarbonate	34	22-32 mmol/L
Glucose	219	65-99 mg/dL
Phosphate	4.4	2.5 to 4.5 mg/dL
Albumin	3.2	3.4 to 5.4 g/dL
Aspartate aminotransferase (AST)	17	15-41 U/L
Alanine aminotransferase (ALT)	27	17-63 U/L
Alkaline phosphatase (ALP)	68	38-126 U/L

## Discussion

Adults with IDD comprise a heterogeneous group of individuals with an underlying health condition, which can impact their health that varies in kind, manifestations, severity, or complexity from other healthy individuals in the community. PWS is one such condition and is the most common form of syndromic obesity in the United States. It is caused by the absence of expression of the paternally active genes in the long arm of chromosome 15 and is equally present in males and females [4]. Besides obesity, patients with PWS often have multiple comorbidities such as growth hormone deficiency, central hypogonadism, hypothyroidism, type 2 diabetes mellitus, sleep apnea, dyslipidemia, cholelithiasis, gastroesophageal reflux, nonalcoholic fatty liver disease, and hypertension [5]. Most individuals with PWS have developmental delay along with mild to moderate intellectual disability and are often dependent upon their caregiver/parents for health care needs [5].

Recent times have seen a steady increase in the rates of chronic conditions among young adults and a proportional increase in the use of multiple medications [6]. Polypharmacy and long-term use of certain medicines are especially prevalent among individuals with IDD [2] leading to a considerable amount of adverse drug reactions [7,8]. In the study by Scheifes et al., authors identified the rate of adverse drug events to be as high as 84% among adults with IDD and concluded these adverse events negatively influence the quality of life [9]. In another study by Erickson et al., authors found adults with IDD to have 2.5 times higher odds of having hospitalization associated with an adverse drug event than the general population suggesting greater severity of adverse drug reactions among adults with IDD [2].

There are several studies and guidelines with recommendations on preventing these adverse events. The UK Stopping Over Medication of People with Learning Disabilities, Autism, or Both campaign (STOMP) is one such attempt to prevent inappropriate psychotropic medication-related polypharmacy among these groups of patients [10]. Deb et al. confirmed improved prescribing behavior among psychiatrists in UK five years following the launch of the STOMP campaign [11]. The 2018 Canadian guidelines have identified family physicians to play the most crucial role in promoting health, overall well-being, and the decision-making process for adults with IDD. Casson et al. found annual comprehensive health assessments (“health checks”) conducted by primary care physicians to be the most comprehensive way of providing care to adults with IDD [12].

Unfortunately, our patient lacked the continuity of care with an established primary care physician, which might have resulted in polypharmacy-induced AKI. Frequent review of medications' effectiveness, indications, adverse drug reactions, or unwanted effects as well as involving a pharmacist whenever possible may help prevent any potential adverse event [3]. Additionally, structured medication review has also been a valuable tool to eliminate drug-related problems and optimize pharmacotherapy in adults with IDD.

## Conclusions

In conclusion, our case illustrates that patients with IDD are at increased risk for polypharmacy-related adverse outcomes, which are preventable medical errors. Hence, continuity of care by an established primary care doctor is critical in managing patients with IDD. Frequent review of medications is necessary to avoid polypharmacy-related adverse events. Clear communication between healthcare providers and caregivers is vital in orchestrating appropriate healthcare delivery to adults with IDD. Additionally, prescribers, pharmacists, and caregivers should be specifically trained to care for patients with IDD.

## References

[1] Bohonowych J, Miller J, McCandless SE, Strong TV (2019). The global Prader-Willi syndrome registry: development, launch, and early demographics. Genes (Basel).

[2] Erickson SR, Kamdar N, Wu CH (2020). Adverse Medication Events Related to Hospitalization in the United States: A Comparison Between Adults With Intellectual and Developmental Disabilities and Those Without. Am J Intellect Dev Disabil.

[3] Sullivan WF, Diepstra H, Heng J (2018). Primary care of adults with intellectual and developmental disabilities: 2018 Canadian consensus guidelines. Can Fam Physician.

[4] Butler MG (1990). Prader-Willi syndrome: current understanding of cause and diagnosis. Am J Med Genet.

[5] Cassidy SB, Schwartz S, Miller JL, Driscoll DJ (2012). Prader-Willi syndrome. Genet Med.

[6] Horace AE, Ahmed F (2015). Polypharmacy in pediatric patients and opportunities for pharmacists' involvement. Integr Pharm Res Pract.

[7] Haider SI, Ansari Z, Vaughan L, Matters H, Emerson E (2014). Prevalence and factors associated with polypharmacy in Victorian adults with intellectual disability. Res Dev Disabil.

[8] O'Dwyer M, Peklar J, McCallion P, McCarron M, Henman MC (2016). Factors associated with polypharmacy and excessive polypharmacy in older people with intellectual disability differ from the general population: a cross-sectional observational nationwide study. BMJ Open.

[9] Scheifes A, Walraven S, Stolker JJ, Nijman HL, Egberts TC, Heerdink ER (2016). Adverse events and the relation with quality of life in adults with intellectual disability and challenging behaviour using psychotropic drugs. Res Dev Disabil.

[10] Branford D, Gerrard D, Saleem N (2019). Stopping over-medication of people with intellectual disability, autism or both (STOMP) in England part 1 - history and background of STOMP. Adv Ment Health Intellect Disabil.

[11] Deb S, Nancarrow T, Limbu B (2020). UK psychiatrists' experience of withdrawal of antipsychotics prescribed for challenging behaviours in adults with intellectual disabilities and/or autism. BJPsych Open.

[12] Casson I, Broda T, Durbin J (2018). Health checks for adults with intellectual and developmental disabilities in a family practice. Can Fam Physician.

